# Extravascular modified lipoproteins: a role in the propagation of diabetic retinopathy in a mouse model of type 1 diabetes

**DOI:** 10.1007/s00125-016-4012-6

**Published:** 2016-06-15

**Authors:** Jeremy Y. Yu, Mei Du, Michael H. Elliott, Mingyuan Wu, Dongxu Fu, Shihe Yang, Arpita Basu, Xiaowu Gu, Jian-Xing Ma, Christopher E. Aston, Timothy J. Lyons

**Affiliations:** 1 Centre for Experimental Medicine, School of Medicine, Dentistry, and Biomedical Science, Queen’s University Belfast, 97 Lisburn Road, Belfast, BT9 7BL Northern Ireland UK; 2 Department of Physiology, University of Oklahoma Health Sciences Center, Oklahoma City, OK USA; 3 Department of Ophthalmology, Dean A. McGee Eye Institute, Oklahoma City, OK USA; 4 Section of Diabetes and Endocrinology, University of Oklahoma Health Sciences Center, Oklahoma City, OK USA; 5 Department of Nutritional Sciences, Oklahoma State University, Stillwater, OK USA; 6 Department of Pediatrics, University of Oklahoma Health Sciences Center, Oklahoma City, OK USA

**Keywords:** Diabetic retinopathy, Dyslipidaemia, Lipoprotein, Oxidised LDL, Pathogenesis, Vascular complications

## Abstract

**Aims/hypothesis:**

We aimed to determine whether plasma lipoproteins, after leakage into the retina and modification by glycation and oxidation, contribute to the development of diabetic retinopathy in a mouse model of type 1 diabetes.

**Methods:**

To simulate permeation of plasma lipoproteins into retinal tissues, streptozotocin-induced mouse models of diabetes and non-diabetic mice were challenged with intravitreal injection of human ‘highly-oxidised glycated’ low-density lipoprotein (HOG-LDL), native- (N-) LDL, or the vehicle PBS. Retinal histology, electroretinography (ERG) and biochemical markers were assessed over the subsequent 14 days.

**Results:**

Intravitreal administration of N-LDL and PBS had no effect on retinal structure or function in either diabetic or non-diabetic animals. In non-diabetic mice, HOG-LDL elicited a transient inflammatory response without altering retinal function, but in diabetic mice it caused severe, progressive retinal injury, with abnormal morphology, ERG changes, vascular leakage, vascular endothelial growth factor overexpression, gliosis, endoplasmic reticulum stress, and propensity to apoptosis.

**Conclusions/interpretation:**

Diabetes confers susceptibility to retinal injury imposed by intravitreal injection of modified LDL. The data add to the existing evidence that extravasated, modified plasma lipoproteins contribute to the propagation of diabetic retinopathy. Intravitreal delivery of HOG-LDL simulates a stress known to be present, in addition to hyperglycaemia, in human diabetic retinopathy once blood-retinal barriers are compromised.

**Electronic supplementary material:**

The online version of this article (doi:10.1007/s00125-016-4012-6) contains peer-reviewed but unedited supplementary material, which is available to authorised users.

## Introduction

Diabetic retinopathy remains a leading cause of new-onset blindness in adults [1], but its pathogenesis is not fully understood. It is known that ‘residual risks’ exist even after controlling for hyperglycaemia [2]. Among these, plasma lipoproteins have been implicated, as supported by numerous clinical and epidemiological studies; see reviews [3–5] and some key studies [6–14]. Most of these studies focus on standard measures of circulating lipids and lipoproteins, and in general, they show associations between ‘dyslipidaemia’, for example elevated plasma LDL-cholesterol or apolipoprotein B (apoB), and the severity of diabetic retinopathy. Some studies have gone further, addressing qualitative (and not just quantitative) changes in plasma lipoproteins, including the extent of LDL modification by oxidation [15]. We have previously reported associations between diabetic retinopathy severity and average lipoprotein particle diameters and the distribution of ‘subclasses’ of HDL, LDL, and VLDL in the Diabetes Control and Complications Trial/Epidemiology of Diabetes Interventions and Complications (DCCT/EDIC) cohort [10]. Also in that cohort, we found that circulating immune complexes containing oxidised LDL (ox-LDL) predicted severe diabetic retinopathy many years later [13]. Overall, however, associations between plasma lipoproteins and diabetic retinopathy, although statistically significant in population studies, are too weak to define individual risk or prognosis, as recently underlined in two detailed reports [14, 15].

We have hypothesised that despite these rather unimpressive associations between plasma lipoproteins and diabetic retinopathy, lipoproteins actually play a central role in diabetic retinopathy progression, but one that is ‘hidden’, occurring in tissue, and only operative after extravasation and modification. In the retina, in contrast to arteries during atherogenesis, leakage from plasma to tissue only occurs if the blood-retinal barriers (BRBs) are compromised. Put simply, plasma lipoprotein levels may be largely irrelevant to the retina if BRBs are intact, and their effects following BRB breakdown may depend more on the extent of leakage than on their plasma concentrations. In support of this concept, we have observed that intra-retinal deposits of apoB-100 (the main protein component of LDL and VLDL), ox-LDL and ox-LDL immune complexes are entirely absent in healthy human retinas, but present in diabetic retinas even before the onset of clinical retinopathy, and thereafter in greater amounts commensurate with diabetic retinopathy severity [16, 17]. Furthermore, we have consistently observed toxicity of LDL (modified in vitro to simulate stresses found in vivo), towards cultured human retinal cells, including capillary endothelial cells, pericytes, retinal pigment epithelial (RPE) cells, and Müller cells, promoting oxidative and endoplasmic reticulum (ER) stresses, inflammation and death [17–24]; ox-LDL is also injurious to neurons [25]. Based on these considerations, we hypothesise that after extravasation into the retinal tissue, lipoproteins become modified by glycation and oxidation and, thus, become cytotoxic. In this way, episodes of initially transient, localised vascular leakage may lead to vicious cycles of chronic pathology.

Rodent models are commonly used in diabetic retinopathy research, but they have limitations. Rodents do not develop the advanced, proliferative retinopathy that afflicts humans [26]. Oxygen-induced retinopathy models in new-born rodents exhibit neovascularisation, but the animals are not a model of diabetes. Regarding a possible role of extravasated lipoproteins in the propagation of diabetic retinopathy, a critical difference between rodents and humans lies in their lipoprotein metabolism: rodents have substantially lower LDL levels than humans and cholesterol is transported primarily in HDL [27]. Therefore, rodent models of diabetes fail to replicate the chronic exposure to extravasated, modified LDL that we have demonstrated in human diabetic retinopathy. In the present study, using intravitreal injection of human LDL (with or without modification), we assessed the effects on retinas in a mouse model of diabetes and in non-diabetic mice.

## Methods

### Animals

Male C57BL/6J mice (7–8 weeks old) were obtained from Jackson Laboratory (Bar Harbor, ME, USA), and housed on a 12/12 h dark-light cycle in a controlled environment (22.5 ± 0.5°C, 50 ± 5% humidity) with access to food and water ad libitum. After at least one week of acclimatisation, animals were randomised into the study by weight. A model of type 1 diabetes was induced with streptozotocin (50–55 mg/kg, daily for 5 days, i.p.; Enzo Life Sciences, Farmingdale, NY, USA); non-diabetic controls received vehicle. Development of diabetes (fasting blood glucose ≥16.7 mmol/l) was confirmed 28 days later, and mice were maintained for eight additional weeks before intravitreal injections. Animals that did not convert to diabetes or that later showed apparent intraocular injection injuries were excluded, but otherwise all animals that consecutively completed the study protocol were included. As far as possible, the ocular outcome assessments described below were performed using a ‘blinded’ study design. The study was approved by the Institutional Animal Care and Use Committee of the University of Oklahoma Health Sciences Center.

### Human lipoprotein preparation

As previously described [19, 22], freshly pooled plasma was obtained from four to six healthy, non-diabetic volunteers aged 20–40 years who were taking neither prescribed medications nor antioxidant vitamins. All donors gave informed consent; the study was approved by the Institutional Review Board of University of Oklahoma Health Sciences Center and was carried out in accordance with the Declaration of Helsinki as revised in 2008. Native LDL (N-LDL; density 1.019–1.063 g/ml) was prepared by sequential ultracentrifugation. To prepare highly-oxidised glycated LDL (HOG-LDL), N-LDL was glycated in 50 mmol/l glucose (72 h, 37°C) under antioxidant conditions (1 mmol/l diethylenetriaminepentaacetic acid (DTPA; Sigma-Aldrich, St Louis, MO, USA), 270 μmol/l EDTA, under nitrogen), dialysed to remove glucose and antioxidants, and then further oxidised in 10 μmol/l CuCl_2_ (24 h, 37°C). After extensive dialysis, protein concentrations were quantified (Pierce BCA assay; ThermoFisher Scientific, Waltham, MA, USA). The preparations were characterised by electrophoresis and tested to be free of endotoxin (Limulus Amoebocyte Lysate assay, Bio-Whittaker, Walkersville, MD, USA).

### Intravitreal administration of LDL

Mice were anaesthetised using ketamine (100 mg/kg i.p.; Henry Schein Animal Health, Dublin, OH, USA) and xylazine (10 mg/kg, i.p.; Vedco, St. Joseph, MO, USA), then received intravitreal administration of 1 μl N-LDL or HOG-LDL (5 μg protein/μl, diluted in PBS), or PBS, using a 36-gauge needle/syringe assembly (World Precision Instruments, Sarasota, FL, USA). Both N-LDL and HOG-LDL were confirmed to be soluble in rat vitreous humour ex vivo. In each animal, one eye was injected with either N-LDL or HOG-LDL, and the other with PBS as a control.

The intraocular kinetics of exogenous LDL is unclear; we aimed to provide a target LDL concentration of approximately 0.5 μg protein/μl, assuming an average vitreous humour volume of 10 μl [28–30]. This approximates the LDL concentration in human plasma. We hypothesise that local ox-LDL concentrations in diabetic retinas may be much higher, considering its non-uniform, patchy distribution [16, 17], as in atherosclerotic plaques [31].

### Electroretinography

Full-field scotopic electroretinography (ERG) was recorded at 3, 7, and 14 days after ocular injection, using established methods [32, 33]. Briefly, after dark adaptation overnight, mice were anaesthetised and maintained on a 37°C heating pad. The pupils were dilated with 2.5% phenylephrine (Paragon Bioteck, Portland, OR, USA) and 1% tropicamide (Bausch & Lomb, Rochester, NY, USA) eye drops. ERGs were recorded bilaterally and simultaneously using gold wire electrodes placed on the cornea, with the reference and ground electrodes positioned at the nasal fornix and the tail, respectively.

### Histology

The eyes were fixed overnight in Perfix (20% isopropanol [vol./vol.] and 2% trichloroacetic acid, 4% paraformaldehyde and 2% zinc chloride [wt/vol]), and placed in 70% ethanol before paraffin embedding. Sagittal sections of 5 μm thickness were stained with haematoxylin and eosin.

### Retinal albumin leakage

Immunohistochemistry of extravasated albumin was used to assess retinal vascular leakage, thus avoiding potential interference from effects of exogenous tracers on BRB function [34, 35]. Retinal sections were deparaffinised and permeabilised with 0.5% (vol./vol.) Triton X-100 for 10 min, followed by incubation with the primary antibody (goat polyclonal anti-albumin, 1:400; Novus Biologicals, Littleton, CO, USA) at 4°C overnight. Alexa Fluor 488-conjugated polyclonal anti-goat IgG (1:400) was used as a secondary antibody (Life Technologies, Carlsbad, CA, USA). The antibodies were used as per the manufacturer’s instructions. DAPI was used for nuclear staining (Life Technologies, Carlsbad, CA, USA). Immunofluorescence was detected under a Nikon E800 microscope (Nikon, Tokyo, Japan), analysed by ImageJ (https://imagej.nih.gov/ij), with the background subtracted. Data were analysed using a modified two sample median test for the distribution of pixel intensities. For each eye, superior and inferior sections were randomly selected and imaged at magnification ×10. The total of all pixel intensity measures from vitreous and retina (from the ganglion cell layer to outer nuclear layer) was used as an index of vascular leakage.

### Retinal flat mounts

For further visualisation of vascular leakage, two labelled dextrans (FITC-dextran, 2000 kD and Texas Red-dextran, 3 kD; 100 μl of 1:1 cocktail; Life Technologies, Carlsbad, CA, USA) were administered by intra-cardiac injection to anaesthetised mice. After 10–15 min, retinas were fixed, flat-mounted, and imaged using an Olympus FluoView FV500 confocal microscope (Olympus, Center Valley, PA, USA).

### Western blots

Retinas were homogenised with RIPA lysis buffer (Santa Cruz Biotechnology, Santa Cruz, CA, USA), with the protein concentration determined (Pierce BCA assay; ThermoFisher). Total protein (30 μg) was resolved by 12% SDS-PAGE and electrophoretically transferred to nitrocellulose membranes (Bio-Rad, Hercules, CA, USA). Membranes were blocked for 1 h with 5% (wt/vol.) non-fat milk in Tris-buffered saline with Tween 20 (TBST), and incubated overnight (4°C) with primary antibodies (Abcam, Cambridge, MA, USA) against glial fibrillary acidic protein (GFAP; 1:5000), vascular endothelial growth factor (VEGF; 1:1000), phosphorylated elF2α (p-elf2α; 1:500), amino acid sequence Lys-Asp-Glu-Leu (KDEL; 1:2000) and Bcl2-associated X protein (BAX; 1:1000) diluted in 5% BSA in TBST, followed by incubation for 1 h at room temperature with horseradish peroxidase (HRP)-conjugated secondary antibody (Vector Laboratories, Burlingame, CA, USA), diluted 1:5000 with 5% (wt/vol.) milk (Bio-Rad). The antibodies were used as per the manufacturer’s instructions. Signals were visualised using the SuperSignal West Dura extended duration substrate kit (ThermoFisher) and quantified by digital image analysis (Syngene, Frederick, MD, USA). Blots were subsequently stripped and re-probed with the β-actin antibody (1:5000; Abcam) for normalisation.

### Immunohistochemistry

Retinal sections were deparaffinised, blocked by 5% (vol./vol.) donkey serum (Sigma-Aldrich) in 1% (vol./vol.) Triton X-100/PBS for 1 h, and incubated overnight at 4°C with the primary antibody (rabbit polyclonal anti-GFAP, 1:1000 and mouse monoclonal anti-KDEL, 1:100; Abcam). Alexa Fluor 555-conjugated anti-rabbit IgG (1:4000) and Alexa Fluor 488-conjugated anti-mouse IgG were used as secondary antibodies (Life Technologies). The nuclei were counterstained with either DAPI (Life Technologies) or propidium iodide (Sigma-Aldrich). Images were captured by a Nikon E800 Epifluoresence microscope.

### Statistics data

Statistics data are expressed as means ± SEM. Statistical analyses were conducted by one-way ANOVA followed by Newman–Keuls post hoc test using Prism 5 (GraphPad, La Jolla, CA, USA). *p* < 0.05 was considered significant.

## Results

### Diabetes sensitised retinas to modified-LDL-induced injury in mice

**Fig. 1:**
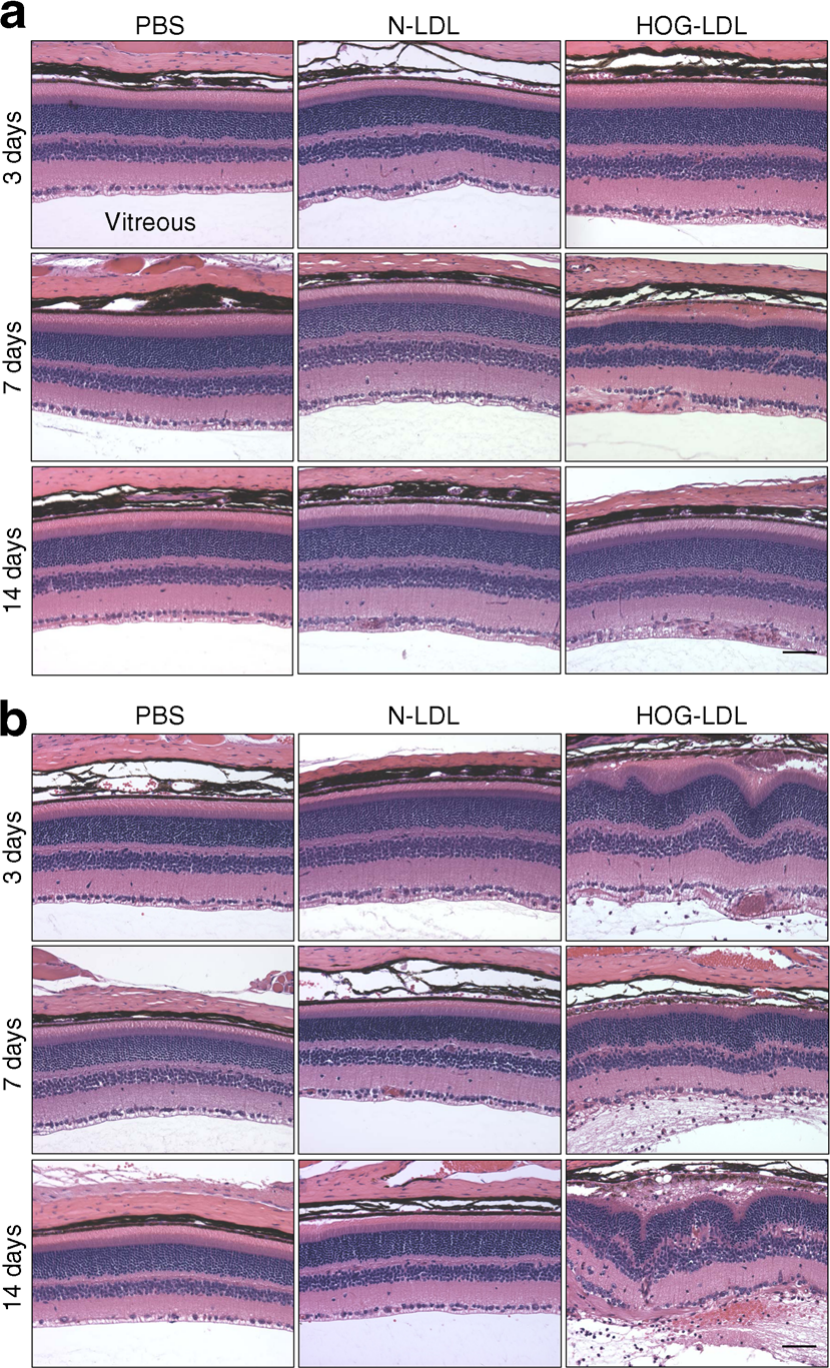
Haematoxylin and eosin stained retinal sections after intravitreal injection of HOG-LDL vs N-LDL and PBS in non-diabetic and diabetic mice. (**a**) In non-diabetic mice, PBS and N-LDL did not affect retinal morphology; HOG-LDL elicited a transient inflammatory response on day 7 in the innermost layer of retina. (**b**) In diabetic retinas, PBS and N-LDL again had no effects, but HOG-LDL caused major structural disruption: marked inflammation and exudation were present by day 3, adjacent to the inner limiting membrane and vitreous; by day 14, injury extended throughout the retina. The rosette-like formations in the outer retina on day 14 reflect the loss of association between neural retina and the RPE layer. Magnification ×20; scale bar 100 μm

Fig. 1 shows representative retinal morphology after intravitreal delivery of HOG-LDL vs N-LDL or PBS in diabetic and non-diabetic mice. N-LDL or PBS had no apparent effects on the retina, regardless of glycaemic status, and diabetic animals had not, as yet, developed any apparent pathology. In non-diabetic mice, HOG-LDL induced a transient, mild infiltration of immune cells in the photoreceptor and ganglion cell layers on day 7, which resolved by day 14 (Fig. 1a). In contrast, in diabetic mice, HOG-LDL triggered extensive inflammation with progressive disruption in all retinal layers (Fig. 1b): on day 3, infiltrating cells were detectable in the ganglion cell layer and vitreous; on day 7, more inflammatory cell infiltrations extended into the vitreous (see electronic supplementary material [ESM] Fig. 1), associated with diffuse photoreceptor damage and swelling of RPE cells; and on day 14, retinal injury became more extensive, with severe disorganisation of retinal architecture. The data suggest that diabetes renders an apparently normal retina highly susceptible to HOG-LDL-induced injury.

### Modified LDL impaired retinal function in diabetic, but not non-diabetic, mice

**Fig. 2:**
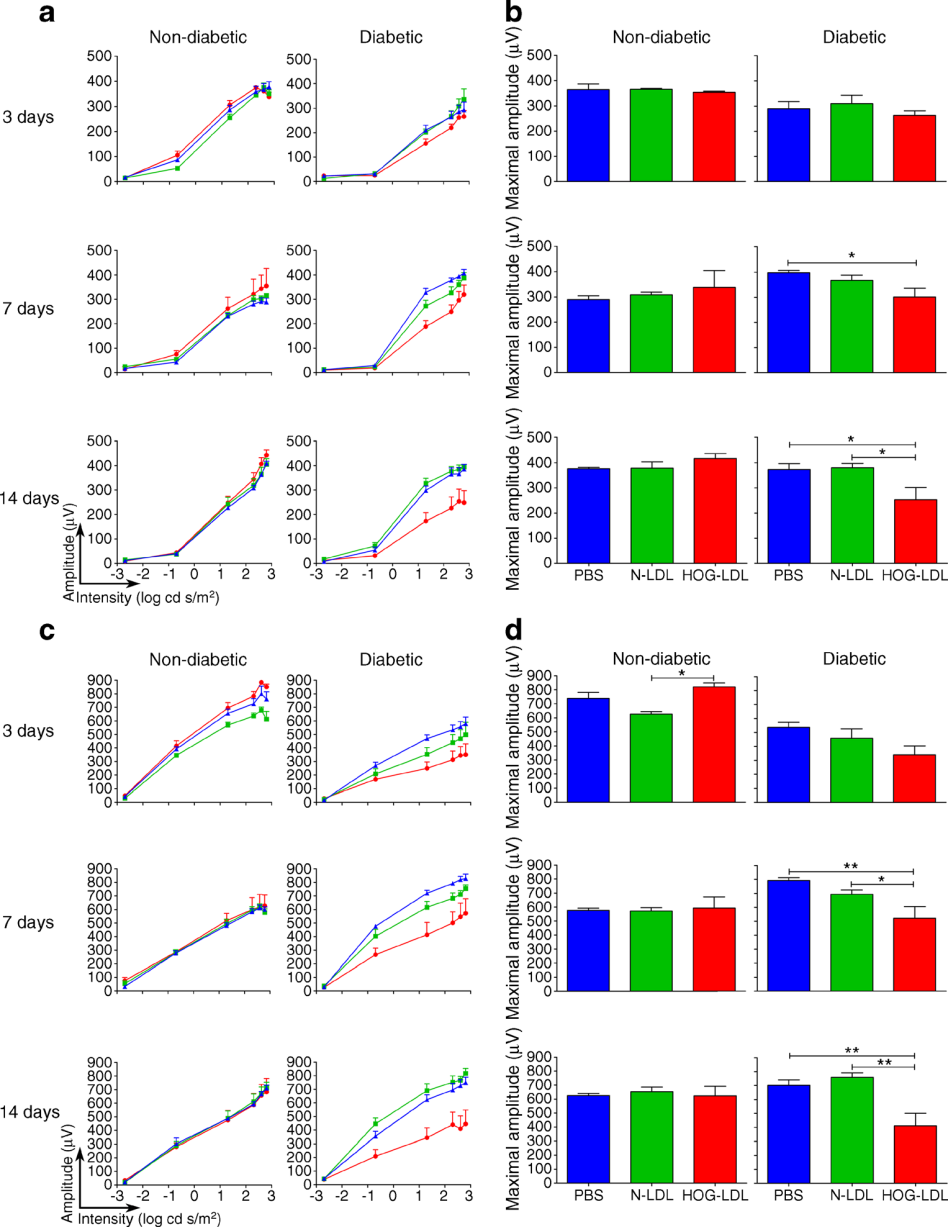
Effects of intravitreal injection of HOG-LDL vs N-LDL and PBS on ERG in non-diabetic and diabetic mice. Scotopic ERGs were recorded at light intensities from −2.7 to +2.8 log_10_ cd s/m^2^. Stimulus-response curves (**a**, **c**) and maximal amplitudes (**b**, **d**) are shown for a-waves (**a**, **b**) and for b-waves (**c**, **d**) after intravitreal administration of HOG-LDL (red), N-LDL (green) or PBS (blue). In non-diabetic mice, HOG-LDL had no effects other than a slightly enhanced b-wave response vs N-LDL on day 3. In diabetic mice, HOG-LDL caused significant reductions in amplitude of a- and b-waves on days 7 (vs PBS) and 14 (vs PBS and N-LDL). Data are presented as means ± SEM, *n* = 5–6 per treatment. *p < 0.05 and **p < 0.01 vs PBS or N-LDL

ERGs were conducted to determine the functional consequences of modified LDL on the retina (Fig. 2). In general agreement with the morphologic findings, ERG responses in diabetic mice after intravitreal administration of PBS or N-LDL were normal and similar to those in non-diabetic animals. In non-diabetic mice, intravitreal delivery of HOG-LDL did not significantly affect the ERG when compared with PBS and N-LDL, but in diabetic animals, HOG-LDL induced significant reductions in both the photoreceptor and second-order neuronal functions at days 7 and 14 vs PBS or N-LDL controls.

### Modified LDL increased retinal vascular permeability in diabetic mice

**Fig. 3:**
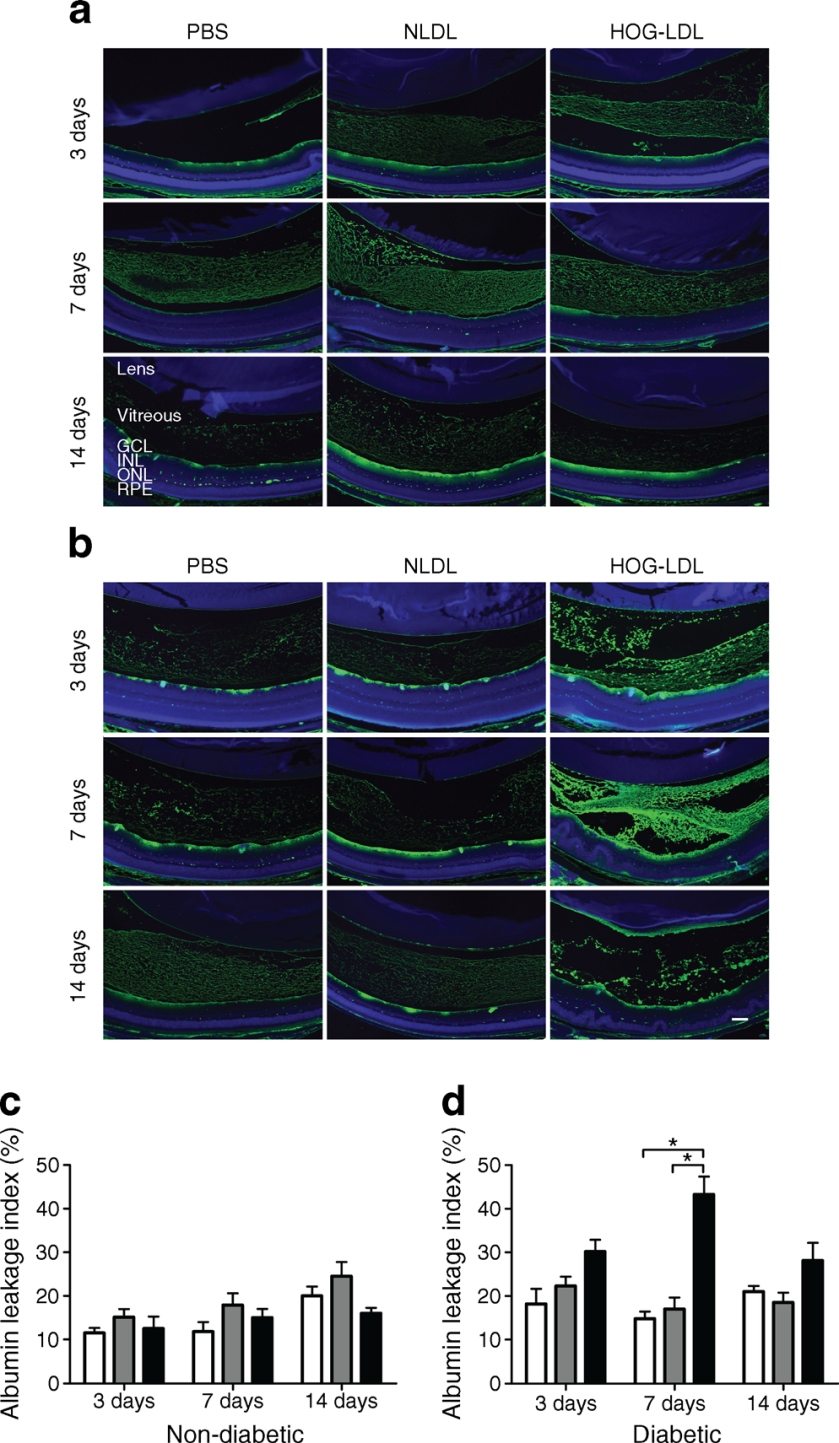
Albumin leakage in the retina and vitreous of non-diabetic and diabetic mice after intravitreal injection of HOG-LDL. Representative immunostaining of albumin (green) and DAPI (blue) on days 3, 7 and 14 post intravitreal injection of HOG-LDL vs N-LDL and PBS in (**a**) non-diabetic and (**b**) diabetic mice. Magnification ×10; scale bar 100 μm. Leakage was quantified in combined retina and vitreous from (**c**) non-diabetic and (**d**) diabetic mice using ImageJ. Data are expressed as mean ± SEM, *n* = 3–4 animals per treatment. *p < 0.05 vs PBS or N-LDL. PBS: white bar; N-LDL: grey bar; HOG-LDL: black bar

**Fig. 4:**
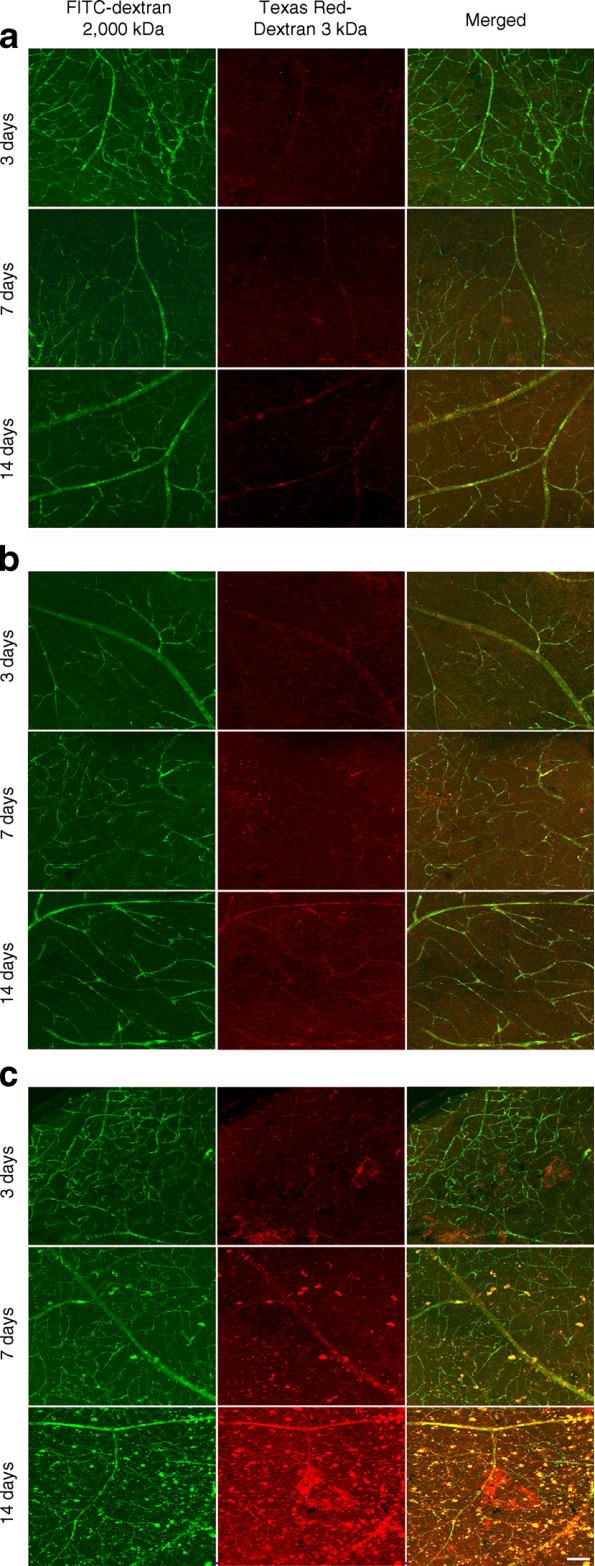
Vascular leakage of fluorescein markers in retinal flat mounts of diabetic mice after intravitreal injection of HOG-LDL. FITC-dextran (2000 kDa, green) and Texas Red-dextran (3 kDa, red) were administered intracardially to anaesthetised diabetic mice 3–14 days after intravitreal injection of (**a**) PBS, (**b**) N-LDL, and (**c**) HOG-LDL. Retinal flat mounts were prepared after 10–15 min. Leakage of both fluorescein markers in the retina of HOG-LDL injected diabetic mice appeared patchy, resembling clinical features of diabetic retinopathy. Magnification ×20; scale bar 100 μm

We employed two techniques to assess the integrity of BRBs. First, we quantified the extravasated endogenous albumin within the retina and vitreous. In non-diabetic mice, albumin staining was observed in the ganglion cell layer only, and there were no significant differences between treatment groups (Fig. 3a, c). In contrast, intravitreal injection of HOG-LDL vs N-LDL or PBS in diabetic mice increased the presence of albumin in the retina and vitreous, reaching statistical significance on day 7 and a trend of increase on days 3 and 14 (Fig. 3b, d). Second, we assessed retinal leakage of two fluorescein markers in retinal flat mounts from diabetic animals (Fig. 4). Again, no apparent leakage of either FITC-dextran (2000 kD) or Texas Red-dextran (3 kD) was observed following PBS or N-LDL administration. In contrast, HOG-LDL resulted in detectable extravasation as early as 3 days, becoming increasingly severe at days 7 and 14, especially with the 3 kD marker. Leakage of both fluoresceins in the retinas of HOG-LDL treated diabetic mice appeared to be scattered and patchy, resembling clinical diabetic retinopathy.

### Modified LDL induced retinal inflammation, ER stress and pro-apoptosis in diabetic mice

**Fig. 5:**
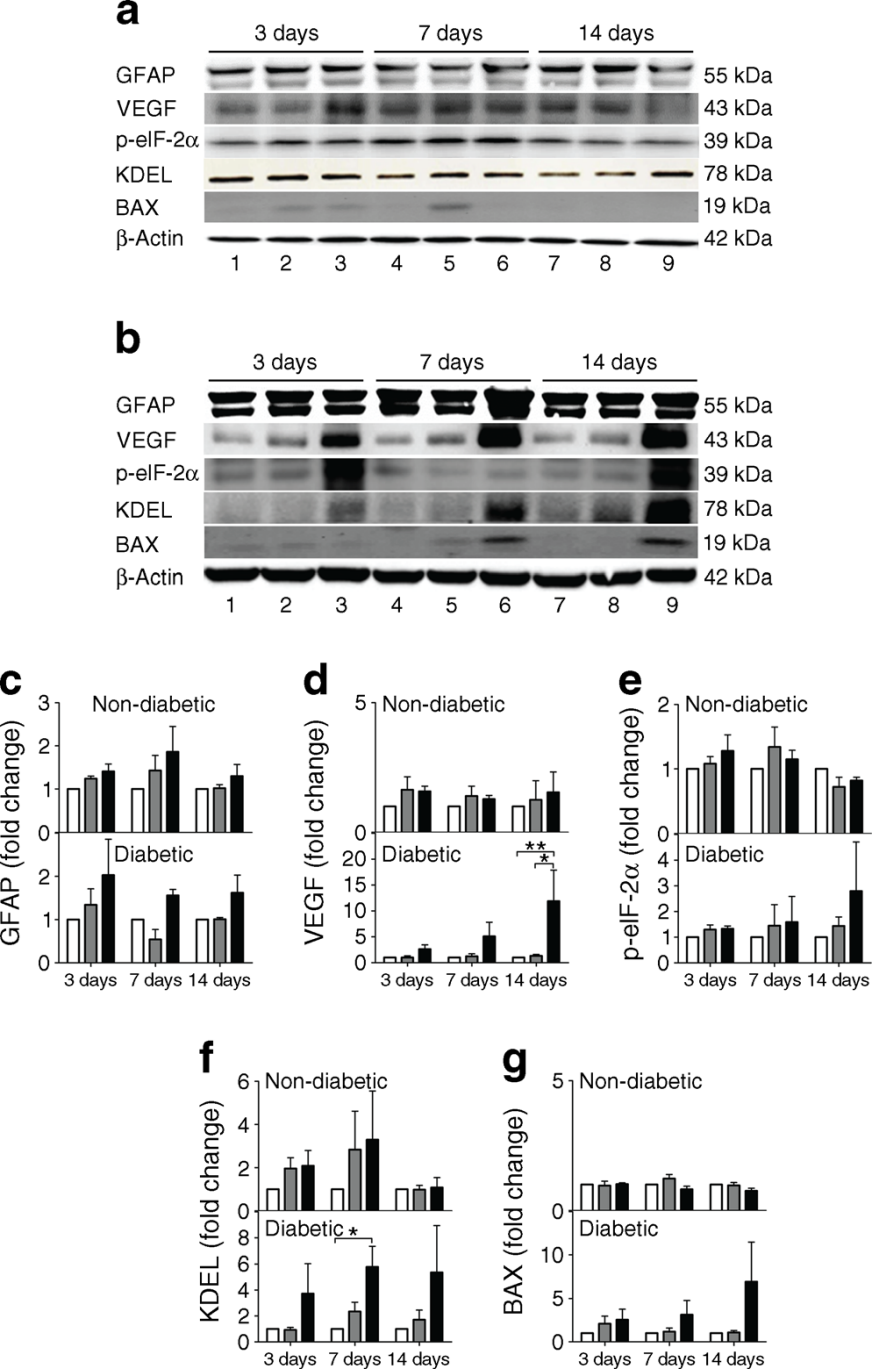
Western blot analyses of retinal biomarkers for inflammation, ER stress and pro-apoptosis after intravitreal injection of HOG-LDL vs N-LDL and PBS in non-diabetic and diabetic mice. Representative western blots for GFAP, VEGF, p-elF2α, KDEL and Bax expression 3–14 days after intravitreal injection of HOG-LDL (lanes 3, 6, 9) vs N-LDL (lanes 2, 5, 8) and PBS (lanes 1, 4, 7) in (**a**) non-diabetic and (**b**) diabetic mice. (**c**–**g**) Quantitative analyses of blots; relative fold change vs PBS controls at respective time points. PBS: white bar; N-LDL: grey bar; HOG-LDL: black bar. Data are expressed as mean ± SEM; *n* = 3–4 per treatment. *p < 0.05 and **p < 0.01 vs PBS or N-LDL

**Fig. 6:**
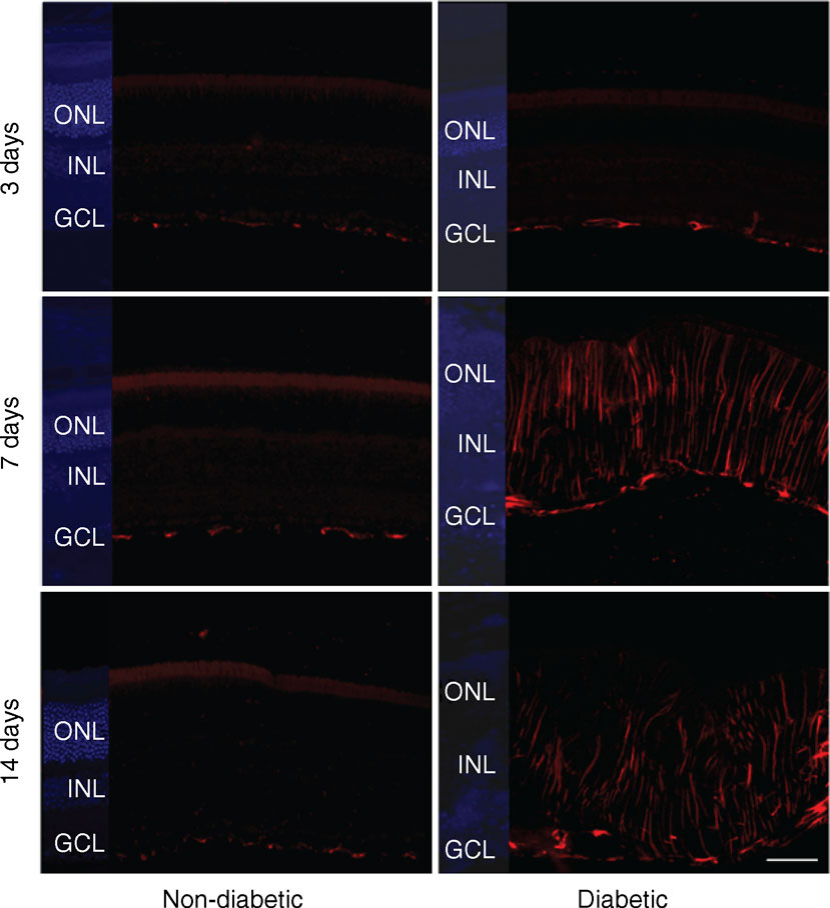
GFAP immunofluorescence in non-diabetic and diabetic retinas after intravitreal injection of HOG-LDL. Extensive glial activation was observed in diabetic, but not in non-diabetic, mouse retinas 7–14 days after HOG-LDL injection, shown as abundant GFAP immunostaining (red) in Müller cell end feet, with radial processes stained intensely throughout inner and outer retina. DAPI (blue) is indicated on the left side of each panel to show the location of the retinal nuclear layers. GCL, ganglion cell layer; INL, inner nuclear layer; ONL, outer nuclear layer. Scale bar: 100 μm

**Fig. 7:**
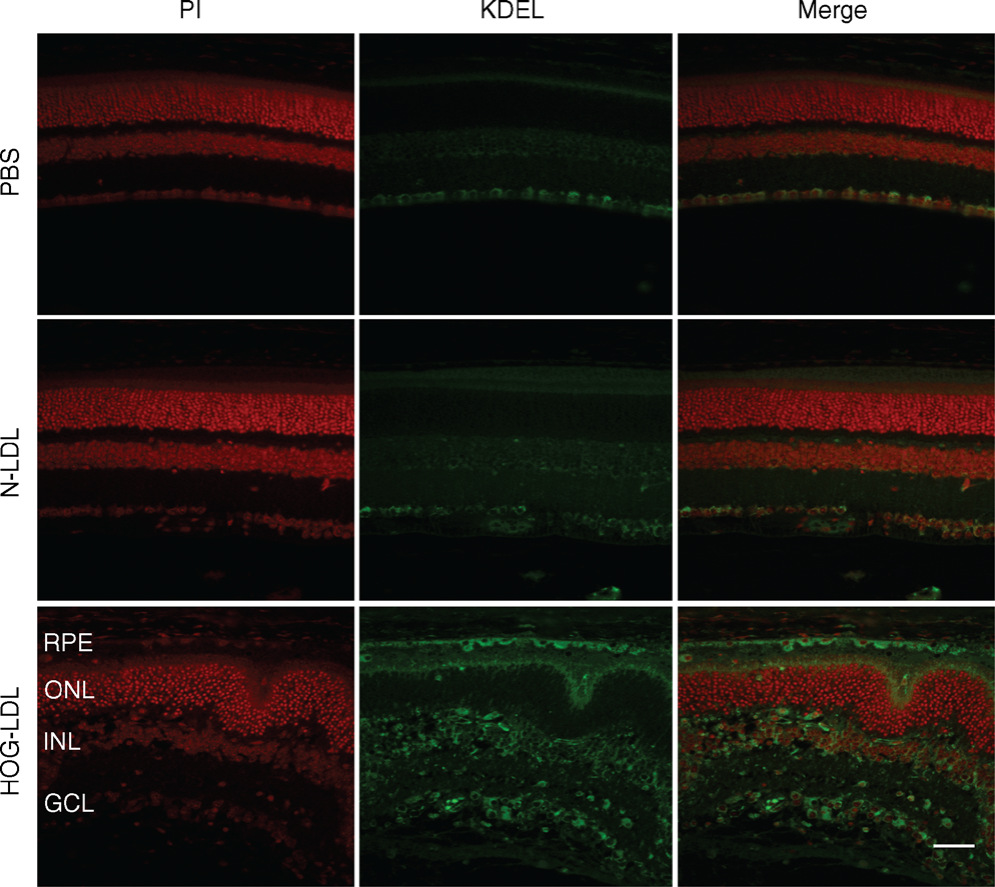
Expression of KDEL in retinal sections of diabetic mice 7 days after intravitreal injection of HOG-LDL. Retinal sections from diabetic mice were stained with antibody against KDEL (green) and nuclei were counterstained by propidium iodide (PI, red). KDEL staining was increased in the ganglion cell layer (GCL), inner nuclear layer (INL), and RPE layer, but not in the outer nuclear layer (ONL), in diabetic retinas after intravitreal injection of HOG-LDL vs N-LDL or PBS. Magnification ×40; scale bar 50 μm

To understand the molecular pathology of modified LDL-induced retinal injury in diabetes, we explored alterations in protein markers for glial activation (GFAP), angiogenesis/vascular permeability (VEGF), ER stress (KDEL and p-eIF2α) and apoptosis propensity (BAX) in mouse retina. HOG-LDL had broadly similar effects to N-LDL in non-diabetic mice (Fig. 5a), but tended to induce retinal expression of GFAP, VEGF, KDEL, p-eIF2α, and BAX in diabetic animals (Fig. 5b), reaching significance in the case of VEGF (see also Fig. 5c–g). Some of the markers, including VEGF, p-eIF2α, KDEL and BAX, also showed an apparent time-dependency, i.e. their retinal levels increased progressively from day 3 to day 14 (Fig. 5d, e, f, g). Immunohistochemistry showed that, in diabetic mice only, HOG-LDL induced GFAP expression in cells that were morphologically characteristic of Müller cells (Fig. 6). HOG-LDL also upregulated KDEL in diabetic retinas, with increased staining in the ganglion cell layer, the inner nuclear layer, and the RPE layer, as compared with PBS or N-LDL (Fig. 7). This suggests that modified LDL injures not only retinal vessels, but also neural and RPE cells, potentially contributing to diabetic retinopathy by affecting multiple retinal cell types.

## Discussion

We evaluated, for the first time, the effects of normal and modified LDL given by intravitreal injection on the diabetic retina. We showed that in the presence, but not in the absence, of short duration diabetes (i.e., 12 weeks), mouse retinas became highly sensitive to the injurious insult of HOG-LDL, but that normal LDL had little effect. Specifically, HOG-LDL altered the retinal architecture and impaired retinal function as measured by ERG, vascular leakage, VEGF expression, inflammation, ER stress, and propensity to apoptosis; effects that replicate some features of human clinical diabetic retinopathy [36]. Thus, these data provide in vivo proof-of-concept that lipoproteins, when extravasated and modified, have important and dire consequences in the propagation of diabetic retinopathy, belying the relatively weak associations between circulating levels and diabetic retinopathy.

Rodents have low endogenous LDL levels, and we considered chronic intravascular infusion of either human or mouse LDL (with or without modification) to be unsatisfactory for both practical and immunologic reasons [37]. Our goal was to simulate the accumulation of intra-retinal modified LDL and to understand its contribution to the pathogenesis of diabetic retinopathy. We chose the intravitreal route to access the retina in a manner analogous to therapeutic approaches in humans. It should be mentioned that, to our knowledge, the endogenous vitreous levels of N-LDL or ox-LDL in diabetic humans have not been documented in the literature, either in the presence or absence of retinopathy. It has been reported that non-diabetic human and bovine aqueous humour contains only HDL, but not LDL [38]. However, in view of our findings of progressive extravasation and modification of LDL in the diabetic retina [16, 17], both N-LDL and modified LDL may exist in the vitreous of diabetic patients.

An important question concerns possible immune responses to human LDL when introduced to the mouse. We consider such effects do not explain the present findings, first because retinal injury was induced by HOG-LDL but not by N-LDL, and second because HOG-LDL only induced retinopathy in diabetic but not in non-diabetic animals. Ocular immune privilege [39] might have a role. However, it should be noted that modified LDL, with its altered immunogenicity, does trigger immune responses in humans, resulting in immune complexes which are implicated in vascular disorders. In the DCCT/EDIC cohort, we found that higher plasma levels of ox-LDL immune complexes predicted risk of diabetic retinopathy in type 1 diabetes [13]. In patients with type 2 diabetes, higher circulating levels of autoantibodies against malondialdehyde-modified apoB-100 were associated with diabetic retinopathy [11]. It is estimated that 95% of ox-LDL in circulation may be immune-complexed [40]. Recently, we reported the presence of ox-LDL immune complexes in human diabetic retinas, and in cell culture; these exhibited greater toxicity than non-complexed ox-LDL towards pericytes [17]. Thus, immune responses to ox-LDL are involved in diabetic retinopathy, but are unlikely to explain the present findings. In the present study, HOG-LDL induced inflammatory cell infiltration in both diabetic and non-diabetic animals, but the response was much more dramatic in the diabetic animals, and, therefore, the effect cannot be attributed to immunologic response to a lipoprotein from a different species.

One intriguing finding from this study is that diabetes appears to ‘prime’ the retina to the injurious effects of HOG-LDL. The dramatic response to HOG-LDL suggests that subclinical changes had already occurred, even though the retina appeared to be normal. The existence of subclinical injury in diabetes agrees with clinical observations; retinas remain apparently normal for over 5 years [41], after which the development and rate of progression of diabetic retinopathy vary widely among individuals [42]. A seminal study by Engerman and Kern [43] showed that in diabetic dogs, poor glycaemic control caused subclinical damage, the progression of which could not be prevented by subsequent good glycaemic control. One plausible explanation for our data is that diabetes (characterised by hyperglycaemia) upregulates CD36 in retinal cells [44], which mediates the toxicity of modified LDL [17]: this is consistent with the observed synergistic effects of glucose and lipids in macrophages via the CD36 receptor in atherosclerotic lesions [45]. Thus the data support a potentially important role of extravasated lipoproteins, in addition to hyperglycaemia, in the propagation of diabetic retinopathy.

Loss of BRB integrity is a critical feature of early diabetic retinopathy [36]. In this study, intravitreal administration of HOG-LDL elicited extensive BRB breakdown in diabetic, but not in non-diabetic mice. Retinal leakage of fluorescein markers appeared scattered and patchy, as is seen in clinical diabetic retinopathy. There was also evidence of injury to the RPE layer, suggesting that both inner and outer BRBs were compromised. These effects were seen even though the LDL-related stress ‘approached’ the retinal vessels from the outside, not from the plasma. They were accompanied by progressively elevated VEGF in the retina over the two week timeframe studied, which likely contributed to the higher vascular permeability. Considering the short duration of this study, the present model simulates only the pre-proliferative changes of diabetic retinopathy. It would be of interest to see whether this increase in VEGF is sustained in the longer term, after repetitive or continuing exposure to HOG-LDL, and if this might induce neovascularisation (as is seen in proliferative diabetic retinopathy of humans) in the rodent model. Taking into account these in vivo data and our earlier observations in human retinal vascular cells [17–24], there is now strong evidence that lipoprotein leakage and modification may cause further loss of BRB integrity, and thus constitute an important positive feedback loop propagating retinal injury.

We also studied some additional biochemical markers in mouse retina that reflect known features of diabetic retinopathy, including glial activation, ER stress and pro-apoptosis [46]. HOG-LDL enhanced these processes in diabetic vs non-diabetic mice. Interestingly, retinal ER stress and BAX increased progressively during the two weeks of observation. Thus, the data are in agreement with our earlier findings in cultured human retinal cells exposed to HOG-LDL [17, 20, 22–24], and with studies in the retinas of diabetic patients [16, 23, 24].

Potential limitations include: (1) intravitreal injection may not accurately mimic the continuous LDL extravasation around retinal vessels; (2) only a single LDL dose was studied, and future studies should define an optimal, pathophysiologically relevant dose range; (3) the variables, including duration of diabetes before LDL challenge and that after injection, require optimisation; and (4) the mechanisms underlying the potentiating effects of HOG-LDL when combined with diabetes require investigation.

In conclusion, our study shows that modified LDL, if present in the retina at early stages of diabetes, significantly accelerates the progression of retinal injury and recapitulates many features of human diabetic retinopathy. It supports the hypothesis that, in addition to hyperglycaemia, and despite rather weak associations between plasma lipoproteins and human diabetic retinopathy, LDL (and presumably other lipoproteins) plays an important but ‘hidden’ role in diabetic retinopathy. Improved means to detect early vascular leakage and ‘glycoxidative/lipoxidative’ damage at the tissue and molecular levels may have value in diabetic retinopathy management. The work also provides feasibility to investigate the relative contribution of modified lipids and hyperglycaemia to diabetic retinopathy using an animal model that employs a simple intravitreal injection approach.

## Electronic supplementary material

Below is the link to the electronic supplementary material.

ESM Fig. 1 (PDF 271 kb)

## Abbreviations

apoB: Apolipoprotein B
BAX: Bcl2-associated X protein
BRB: Blood-retinal barrier
EDIC: Epidemiology of Diabetes Interventions and Complications
ER: Endoplasmic reticulum
ERG: Electroretinography
GFAP: Glial fibrillary acidic protein
HOG-LDL: Highly-oxidised, glycated LDL
KDEL: Amino acid sequence Lys-Asp-Glu-Leu
N-LDL: Native LDL
ox-LDL: Oxidised LDL
RPE: Retinal pigment epithelium
VEGF: Vascular endothelial growth factor
