# Supplementary material for: Extravascular modified lipoproteins: a role in the propagation of diabetic retinopathy in a mouse model of type 1 diabetes
Source: Diabetologia. 2016 Jun 15;59:2026–35. doi: 10.1007/s00125-016-4012-6 (PMC4969344; doi:10.1007/s00125-016-4012-6)

Electronic Supplementary Material (ESM) Figure 1. High magnification (100x) representative images of inflammatory cell infiltration after intravitreal injection of HOG-LDL in diabetic C57BL/6J mice.

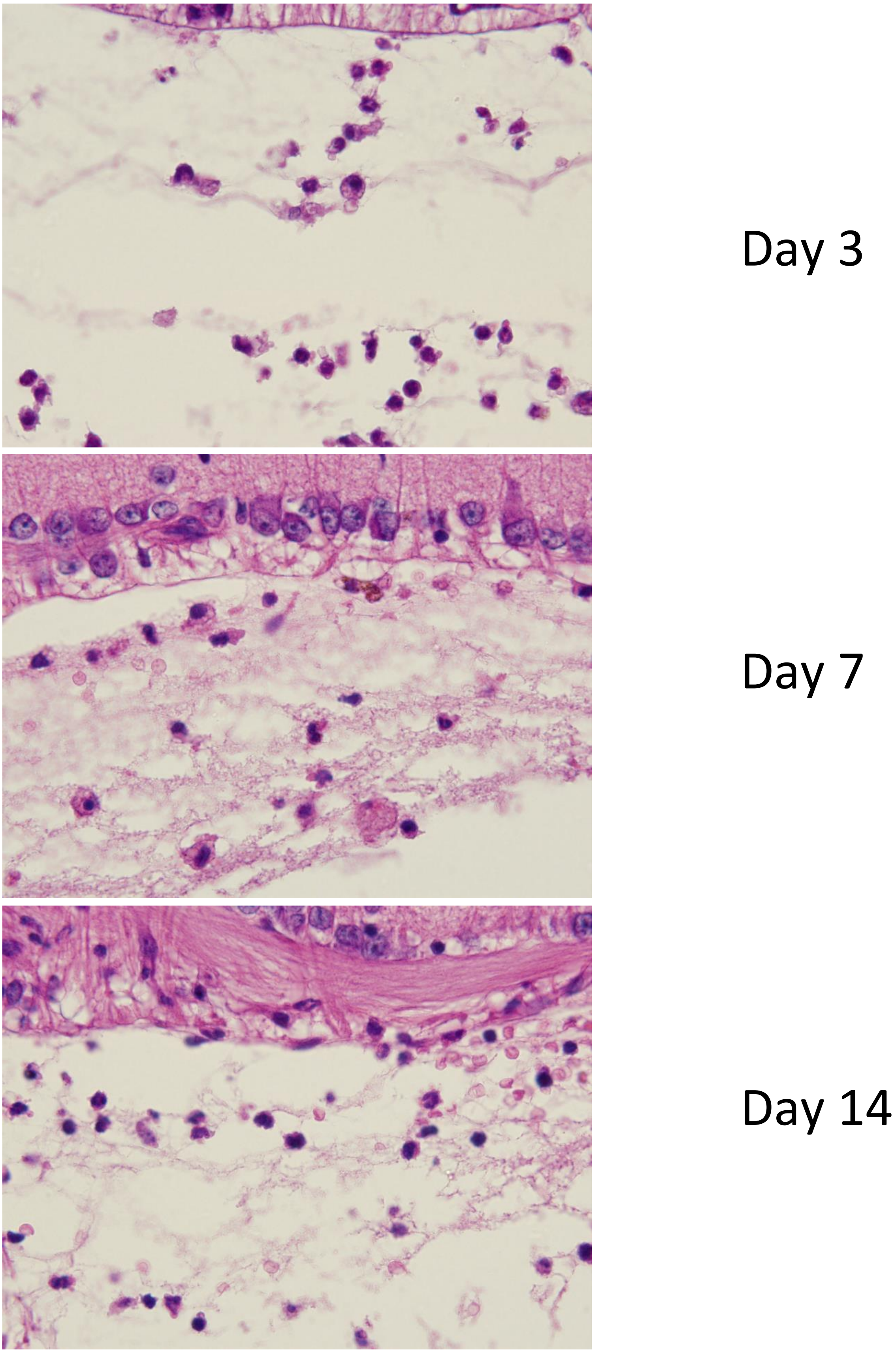

Supplement: Supplementary file 1 — (PDF 271 kb) [file 125_2016_4012_MOESM1_ESM.pdf]
